# Changes in Weight and Substrate Oxidation in Overweight Adults Following Isomaltulose Intake During a 12-Week Weight Loss Intervention: A Randomized, Double-Blind, Controlled Trial

**DOI:** 10.3390/nu11102367

**Published:** 2019-10-04

**Authors:** Helen Lightowler, Lisa Schweitzer, Stephan Theis, Christiani Jeyakumar Henry

**Affiliations:** 1 Oxford Brookes Centre for Nutrition and Health, Department of Sport, Health Sciences and Social Work, Faculty of Health and Life Sciences, Oxford Brookes University, Gipsy Lane Campus, Headington, Oxford OX3 0BP, UK; p0073016@brookes.ac.uk; 2 BENEO-Institute, BENEO GmbH, Wormser Straße 11, 67283 Obrigheim/Pfalz, Germany; lisa.schweitzer@beneo.com (L.S.); stephan.theis@beneo.com (S.T.); 3 Clinical Nutrition Research Centre (CNRC), Singapore Institute for Clinical Sciences (SICS), Agency for Science, Technology and Research (A*STAR) and National University Health System, Centre for Translational Medicine, 14 Medical Drive #07-02, MD 6 Building, Yong Loo Lin School of Medicine, Singapore 117599, Singapore

**Keywords:** isomaltulose, glycemic index, overweight, Palatinose™, weight management, substrate oxidation, diet

## Abstract

Low-glycemic compared to high-glycemic diets have been shown to improve metabolic status and enhance fat oxidation. The randomized, double-blind, controlled intervention study aimed to evaluate the effects of an energy-reduced diet containing isomaltulose (ISO, Palatinose™) versus sucrose (SUC) on body weight loss. Sixty-four healthy overweight/obese adults were allocated to consume either 40 g/day ISO or SUC added to an energy-reduced diet for 12 weeks. Anthropometric measurements, body composition, and energy metabolism were assessed at baseline and after 4, 8, and 12 weeks. Fifty participants (age: 40.7 ± 11.7 y; BMI: 29.4 ± 2.7 kg/m²) completed the study. During the 12 weeks, both groups significantly lost weight (*p* < 0.001), which was more pronounced following ISO (−3.2 ± 2.9 vs. −2.1 ± 2.6 kg; *p* = 0.258). Moreover, for participants in the ISO group, this was accompanied by a significant reduction in fat mass (ISO: −1.9 ± 2.5, *p* = 0.005; SUC: −0.9 ± 2.6%, *p* = 0.224). The overall decrease in energy intake was significantly higher in the ISO compared to that in the SUC group (*p* = 0.022). In addition, breakfast containing ISO induced a significantly lower increase in postprandial respiratory quotient (RQ) (mean incremental area under the curve (iAUC)_2h_ for ISO vs. SUC: 4.8 ± 4.1 vs. 6.9 ± 3.1, *p* = 0.047). The results suggest that ISO in exchange for SUC may help to facilitate body weight reduction, lower postprandial RQ associated with higher fat oxidation, and reduce energy intake.

## 1. Introduction

Excessive energy intake over a prolonged period has been associated with obesity due to the inability of human metabolism to counteract energy imbalance. One regulating factor for energy homeostasis and body weight is substrate utilization where, under healthy conditions, it is largely influenced by carbohydrates and fats [1,2,3,4]. Depending on the macronutrients present, metabolism is flexible to shift substrate utilization from one substrate to the other [2,5]. Accordingly, the fasting state is mainly characterized by fat oxidation, whereas during the postprandial state, after a mixed meal ingestion, carbohydrate oxidation dominates. Conversely, in overweight and obese individuals, substrate utilization has been observed to be altered and is characterized by decreased fat oxidation [6]. This alteration has been linked to fat accumulation in skeletal muscle and organs as well as insulin resistance [2,7]. In contrast, an increase in fat oxidation has been shown to be associated with accelerated weight loss and improved weight management [8].

Previous research has shown that the glycemic index (GI) value of foods has a positive effect on postprandial substrate oxidation. Numerous studies have demonstrated higher postprandial fat oxidation with low GI (LGI) compared to high GI (HGI) meals, both during rest and exercise [9,10,11,12,13]. The proposed mechanism behind this is the attenuation of blood glucose levels and decreased insulin secretion with LGI compared to HGI foods [2,13,14]. Insulin is known to strongly suppress fat oxidation by inhibiting lipolysis [15] and may be one of the key factors not only for regulating substrate metabolism but also for managing body weight. Previous studies have shown that when comparing LGI and HGI diets, an improvement in insulin resistance and other metabolic risk factors was attributed to the consumption of LGI foods [16,17,18,19]. In addition, a greater weight and fat mass loss was seen in overweight and obese subjects who were on a LGI diet [20]. This may be due to increased fat oxidation and prolonged satiety after LGI compared to HGI diets [8,21]. Therefore, the continuous ingestion of an LGI carbohydrate as a replacement for higher glycemic carbohydrates administered within the course of a normal, yet energy-reduced diet, may help to manage body weight in overweight/obese individuals. 

The disaccharide isomaltulose (ISO; Palatinose™) is an isomer of sucrose (SUC) linking glucose and fructose by an α-1,6-glycosidic bond. The hydrolysis of ISO by the sucrase–isomaltase complex is slower, yet complete [22,23], which highlights the use of ISO as a low glycemic, slow-release sugar alternative (GI ISO vs. SUC: 32 vs. 64 [22]). 

The aim of this study was to assess body weight and body composition changes in overweight and obese individuals consuming an energy-reduced diet containing foods with either 40 g/day ISO or SUC for a 12-week period. In addition, postprandial substrate oxidation was determined. It was hypothesized that the continuous replacement of SUC by ISO would support body weight loss and favorably impact body composition due to increased fat oxidation.

## 2. Materials and Methods 

### 2.1. Study Population

Sixty-four healthy overweight and obese adults (age: 18–60 y; BMI: 25–35 kg/m²) who were motivated to lose weight were recruited via posters and flyers around Oxford Brookes University. Exclusion criteria were the presence of any acute and chronic diseases and the use of medications which interfere with body composition, appetite, satiety or food intake. Participants who were restrained eaters, assessed by Stunkard’s dietary restraint questionnaire [24], were also excluded. 

### 2.2. Study Design

The monocenter intervention was a double-blinded, placebo-controlled, randomized trial conducted between April 2009 and June 2010 at the Functional Food Centre at Oxford Brookes University. Test products were provided by BENEO GmbH as single encoded portions. Blinding was maintained by the use of number labelled products to ensure participants and the data analyzers were unaware of whether they consume ISO or SUC. The researchers at Oxford Brookes University were also unaware of the test or control assignment of the participants. The unblinding was only made after statistical analysis of the data.

Using a parallel design, participants were randomly allocated to the test group (ISO) or control group (SUC) via a random order generator (Department of Psychology, Oxford Brookes University) and were instructed to consume a diet containing foods with either 40 g/day ISO or SUC for 12 weeks. Outcome measures were assessed at baseline, weeks 4 and 8, and at the end of the intervention (week 12). 

The study was carried out in accordance with the Declaration of Helsinki, and the protocol was approved by the University Research Ethics Committee of Oxford Brookes University (UREC Registration No: 090382). Written informed consent was obtained from each participant before commencing the study. The trial was registered at clinicaltrials.gov (NCT03652207).

### 2.3. Dietary Intervention

During the 12 weeks, participants were instructed to consume an energy-reduced diet (~1700 kcal; Table S1). The diet was controlled for meals containing ISO or SUC, which were breakfast, mid-morning snack, lunch, and afternoon snack. The daily dosage of 40 g SUC (or ISO) in the context of an ~1700 kcal energy-reduced diet was chosen on the basis of existing advice on daily sugar intake. To enhance food variety as well as adherence to the protocol, participants were allowed to choose their own dinner according to healthy eating guidelines based on the UK Eatwell Guide [25]. The guidelines were introduced to the participants before commencing the study. All test products including ISO and SUC were provided by BENEO GmbH (Mannheim, Germany) as single, encoded portions. 

### 2.4. Data Collection

On the study visits at baseline and after 4, 8, and 12 weeks, participants were instructed to come to the study center after an overnight fast of 10–12 h. 

#### 2.4.1. Anthropometrics and Body Composition

In each participant, all parameters were measured by the researchers in the fasted state. Body weight was measured to the nearest 0.1 kg using a scale (Tanita BC-418 MA; Tanita UK Ltd., Yiewsley, UK), and height was recorded by a stadiometer (Seca Ltd., Birmingham, UK). BMI was calculated using the standard formula: weight (kg)/height squared (m²). Waist circumference (WC) was assessed using a measuring tape at midway between the lowest rib margin and iliac crest [26] and recorded to the nearest 0.1 cm. Body composition, i.e., fat mass (FM) and fat-free mass (FFM), was measured using air-displacement plethysmography (BodPod, Life Measurements Inc., Concord, MA, USA). 

#### 2.4.2. Energy Intake (EI)

Before study visits, the researchers asked each participant to complete weighed food records over three consecutive days (two weekdays and one weekend day) to monitor their compliance to the protocol. The calculation of food intake was determined by the researchers using the Dietplan6 nutrition analysis software package (Forestfield Software Ltd., Horsham, UK). In order to facilitate compliance, participants were requested to return empty test and control packaging and were told to do this for recycling purposes. 

#### 2.4.3. Energy Metabolism

Energy metabolism was calculated by measuring CO2 production and O2 consumption continuously for 30 minutes after an overnight fast using indirect calorimetry (Deltatrac Metabolic Monitor, Datex Instruments, Helsinki, Finland). All measurements were carried out under standardized conditions: early in the morning, with the participant at complete rest in a thermos-neutral environment (24–26 °C), and at least 12 h after the last meal. Participants were asked to lie completely still during the measurements. 

Resting energy expenditure (REE) was calculated from the 30-min measurement in the fasting state using the Weir equation [27]. To obtain postprandial energy metabolism, i.e., respiratory quotient (RQ) and substrate oxidation, continuous gas exchange was determined after breakfast at all study visits. Standardized breakfast on these study visits was supplemented by a fruit drink containing additional 20 g ISO or SUC, i.e., participants consumed 364 kcal and 30 g ISO or SUC in total. Volumes of CO2 and O2 were measured every 30 minutes for 10 minutes over a period of 2 h. Carbohydrate oxidation (CO) and fat oxidation (FO) at baseline, after 30, 60, 90, and 120 min were calculated according to the Frayn equation [28]. Nitrogen excretion was estimated assuming that urinary nitrogen excretion rate was negligible. Energy expenditure from CO or FO was calculated by multiplying the individual energy expenditure data (kcal) with individual percentage of specific substrate oxidation. To assess total postprandial CO and FO, 2-hour total area under the curve (AUC) was calculated using the trapezoidal rule [29]. 

#### 2.4.4. Other Parameters 

Blood pressure, i.e., systolic and diastolic, was assessed in the fasting state according to standard guidelines. Measurement was conducted on the left arm after participants had rested for 5 minutes using an automated sphygmomanometer (UA-767, A&D Instruments Ltd., Abingdon, UK). 

Fasting blood samples were obtained. Blood glucose was measured using HemoCue Glucose 201+ analyzer (HemoCue Ltd., Dronfield, UK). Total cholesterol and triglycerides were analyzed using a dry chemical analyzer (Reflotron®Plus, Roche, DE).

### 2.5. Statistical Analysis

The sample size calculation was carried out for the primary outcome body weight loss. Based on an α of 0.05 (two-sided), β of 0.2, and an effect size of 1 kg (SD ± 1.5 kg), a minimum of 36 participants was necessary to detect significant differences between the groups [30]. In the current study, final analysis was performed in 50 participants who completed the study.

Data were analyzed using the SPSS version 24.0 (SPSS Inc., Chicago, IL, USA). Data were presented as mean ± SD. Prior to statistical analysis, the normality of the data was tested using the Shapiro–Wilk statistic. For assessment of differences between groups, the independent sample t-test (for parametric data) or Mann–Whitney U test (for nonparametric data) was performed. The repeated measures ANOVA test (for parametric data) or the Friedman test (for nonparametric data) was used to assess the impact of the intervention within each group. Post-hoc analysis was performed with the Bonferroni correction for parametric data and the Wilcoxon signed-rank test for nonparametric data. Statistical significance was set at *p* < 0.05 for all tests, with the exception of Wilcoxon signed-rank tests (where required), which were conducted with a Bonferroni correction applied, resulting in a significance level set at *p* < 0.008.

## 3. Results

### 3.1. Baseline Characteristics

**Figure 1:**
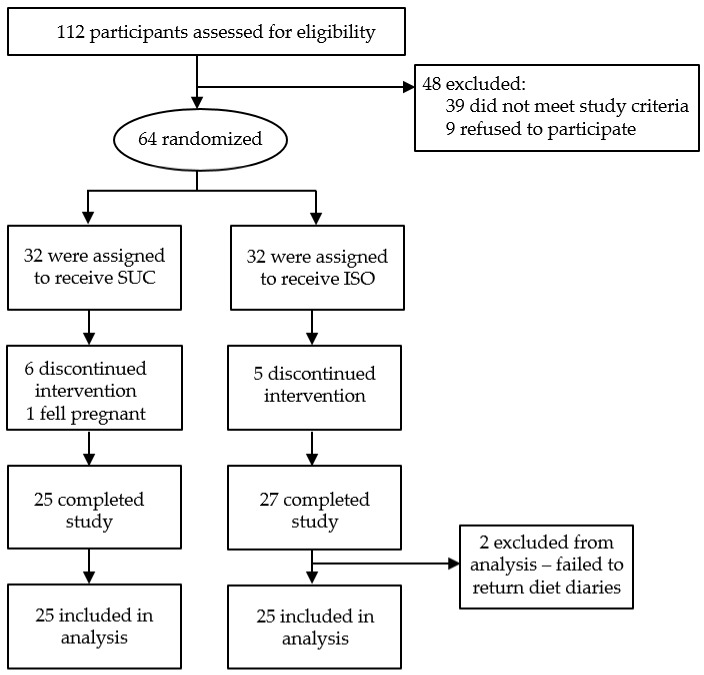
Flow chart of participant numbers. ISO, isomaltulose; SUC, sucrose.

Initially, 112 participants were screened for eligibility, from whom 48 were excluded. Reasons for exclusion were solely based on predefined inclusion/exclusion criteria with the main reason being nutritional status, i.e., BMI. In addition to this, also restrained eating behavior, the age limit of 60, and medications strongly suggested to impact appetite were reasons for excluding the persons in the courses of screening procedure. Of the 64 recruited participants, 11 withdrew from the study and a further three participants were excluded. In total, 50 participants (41 females, 9 males) completed the intervention, 25 participants in each group (Figure 1). 

The age range of the participants who completed the study was 19–59 years, and the initial mean BMI was 29.4 ± 2.7 kg/m². Except for gender and energy intake, participants in both groups were well matched with regard to their baseline characteristics as there were no significant differences between parameters (*p* > 0.05; Table 1). 

### 3.2. Anthropometrics and Body Composition

#### 3.2.1. Body Weight, BMI, and Waist Circumference

**Figure 2:**
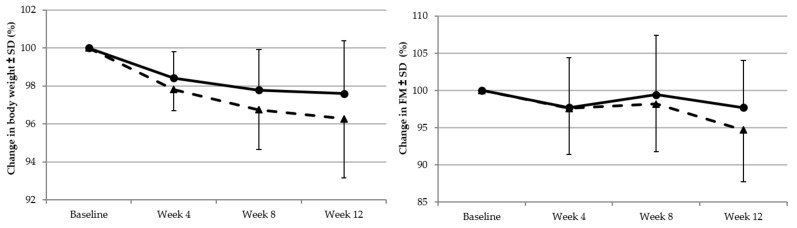
Baseline adjusted mean body weight and body fat changes from baseline at weeks 4, 8, and 12 for the participants consuming isomaltulose (triangles, dotted lines) or sucrose (circles, solid line).

The Friedman test showed that body weight was significantly reduced at 12 weeks compared to baseline in both the ISO group (*p* < 0.001) and the SUC group (*p* < 0.001; Table 1). In both groups, the phase of maximum weight loss occurred during baseline and week 4, which was 1.8 ± 1.0 kg and 1.4 ± 1.3 kg for participants in the ISO and SUC group, respectively. Although the mean rate of weight loss decreased thereafter, participants in both groups continued to lose weight, with a faster rate of weight loss in the ISO group compared to that in the SUC group (Figure 2). The overall weight change from baseline to week 12 was −3.2 ± 2.9 kg and −2.1 ± 2.6 kg for ISO and SUC, respectively (*p* = 0.258; data not shown). 

Although WC declined in both groups, no significant changes in waist circumference were seen at 12 weeks compared to baseline in the ISO group (*p* = 0.347) or the SUC group (*p* = 0.511; Table 1). The overall WC change from baseline to week 12 was −3.3 ± 11.7 cm and −2.1 ± 7.4 cm for ISO and SUC, respectively (*p* = 0.666).

#### 3.2.2. Fat Mass and Fat Free Mass

During the intervention, there was a positive shift in body composition favoring FFM. At the end of the intervention, percentage of FFM (%FFM) was significantly higher compared to baseline in the ISO group (*p* = 0.007; Table 1), whereas for SUC, the increase in %FFM was not significant (*p* = 0.088). The overall increase in %FFM from baseline to week 12 was 2.0 ± 2.4% and 0.9 ± 2.6% for ISO and SUC, respectively (*p* = 0.127).

A reduction in percentage FM (%FM) was observed for both groups (Figure 2), which was significant only for participants who consumed ISO (*p* = 0.005; Table 1). Fat mass reduction with ISO accounted for −1.9 ± 2.5%, which is approximately 1% higher compared to that with the SUC group. The overall decrease in %FM from baseline to week 12 was −1.9 ± 2.5% and −0.9 ± 2.6% for ISO and SUC, respectively (*p* = 0.169).

### 3.3. Energy Intake and Metabolism

#### 3.3.1. Energy and Macronutrient Intake

Data from 3-day food records indicated good compliance by participants to the study diet. The Friedman test showed that there was a significant decrease in EI at 12 weeks compared to baseline in both groups (*p* < 0.001; Table 1). In the ISO group, there was a significant decrease in EI already at weeks 4 and 8 compared to baseline (*p* < 0.008), while in the SUC group, there was a significant decrease compared to baseline only at week 8 (*p* < 0.008). Although mean EI at baseline was significantly higher for ISO compared to that for SUC (*p* = 0.012; Table 1), at all other visits, EI did not differ between the groups. The overall decrease in EI at week 12 was significantly higher in the ISO group compared to that in the SUC group (−544 ± 470 vs. −283 ± 279 kcal/d, *p* = 0.022, data not shown).

Macronutrient intakes were significantly higher at baseline for ISO compared to those for SUC (Protein: *p* = 0.002, 91.8 ± 25.3 g/day and 71.8 ± 15.9 g/day, respectively; fat: *p* = 0.041, 87.9 ± 25.3 g/day and 73.5 ± 22.2 g/day, respectively; carbohydrate: *p* = 0.041, 262.5 ± 60.7 g/day and 232.1 ± 56.0 g/day, respectively; Table S2). There was a significant difference in fat intake at weeks 4, 8, and 12 compared to baseline in both the ISO group (*p* < 0.001) and the SUC group (*p* < 0.001). Furthermore, there was a significant difference in protein at week 12 compared to baseline in the ISO group (*p* = 0.047). There were no significant within-group differences in overall changes in macronutrient intakes in either the ISO or SUC group (Table S3). However, the change in protein intake from baseline to week 12 was significantly greater in the ISO compared to that in the SUC (*p* = 0.005).

#### 3.3.2. Energy Expenditure and Substrate Oxidation

REE did not differ significantly between the groups at baseline or any other visit (Table 1). For ISO, although REE at week 12 did not differ from week 8, REE at week 12 was significantly lower compared to baseline (*p* = 0.007) and week 4 (*p* = 0.007; Table 1). In addition, only for ISO, there was a significant decrease in postprandial EE (*p* = 0.002), whereby this difference was seen between week 12 and baseline (*p* = 0.011). 

**Figure 3:**
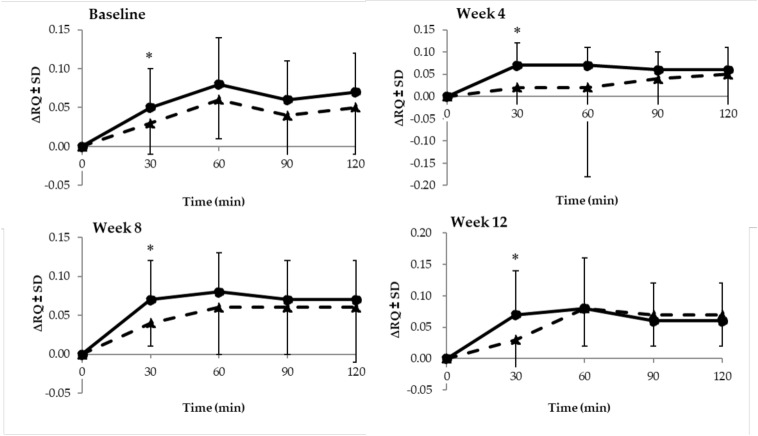
Change in respiratory quotient (RQ) (∆RQ) from fasting state to 30, 60, 90, and 120 minutes after consumption of the test breakfast with isomaltulose (triangles, dotted lines) and sucrose (circles, solid line) at all four study visits. * Independent sample t-test: *p* < 0.05.

**Figure 4:**
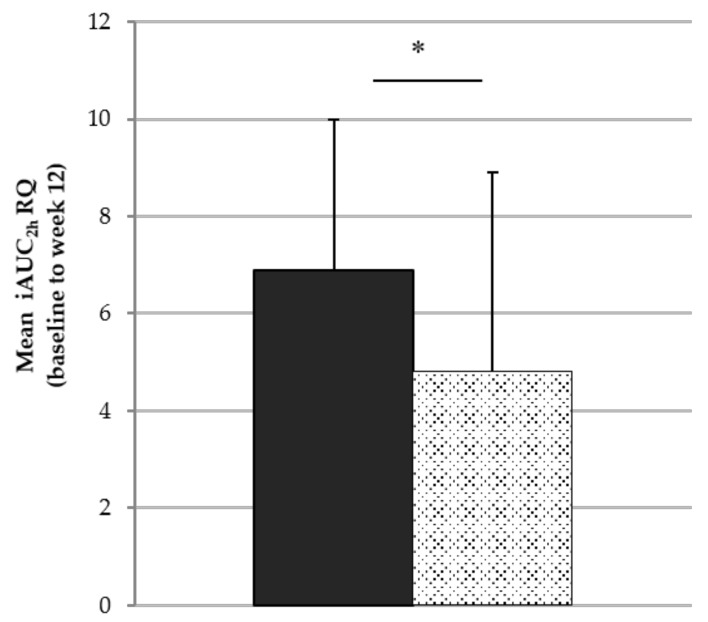
Mean iAUC for RQ of the visits at baseline and weeks 4, 8, and 12 for isomaltulose (grey bar) and sucrose (black bar) considering 2 h post-breakfast consumption (0–120 min). * Independent sample t-test: *p* < 0.05.

In terms of the RQ change after breakfast (0–120 min), a lower increase in RQ was seen with ISO compared to that with SUC (*p* = 0.348; Table 1). For each study visit, there was a significant difference at 30 minutes post-breakfast (*p* < 0.05; Figure 3). RQ for ISO remained lower during the subsequent postprandial state at weeks 0, 4, and 8, although the difference did not reach statistical significance (*p* = 0.374). The breakfast containing ISO induced a significantly lower increase in postprandial RQ (mean iAUC_2h_ for ISO vs. SUC: 4.8 ± 4.1 vs. 6.9 ± 3.1, *p* = 0.047; Figure 4).

Total AUC for substrate oxidation showed a higher CO with SUC when compared to ISO at all four study visits, varying significantly at week 4 (*p* = 0.035, Table 1). In contrast, irrespective of the study visit, FO was higher after breakfast containing ISO compared to that containing SUC (*p* > 0.05; Table 1).

### 3.4. Other Parameters and Adverse events

Blood pressure (systolic, diastolic), blood lipids (total cholesterol, triglycerides), and fasting blood glucose were within a normal range at baseline and during the intervention (Table S4). No serious adverse events were reported in any participants. One participant (2%) reported an upset stomach on the days she consumed cheese sandwiches for lunch. Study diet as well as carbohydrates were overall well tolerated.

## 4. Discussion

One major finding of our study was that the consumption of ISO compared to that of SUC was more effective at promoting weight loss. This finding was supported by a more favorable change in body composition, whereby a larger decrease in %FM was observed. 

As the mean weight loss with ISO compared to that with SUC was not only higher at the end of intervention but also at each single visit compared to the prior visit, this suggests a weight loss-maintaining effect exerted by ISO. The positive shift in substrate oxidation with ISO in favor of fat oxidation may be attributed to its low glycemic and insulinemic effects [12,23]. Therefore, improvements in both body weight and body composition were observed in the participants. Evidence suggests that overweight and obesity corresponds with a decreased fat oxidation accompanied by an increased flux of fatty acids through cell membranes [7]. The concomitant fat accumulation within skeletal muscle and organs, in turn, is associated with the development of insulin resistance as well as cardio-metabolic risk factors [2,6]. To date, there is a broad consensus indicating that an increased fat oxidation leads to an improvement in insulin resistance, muscle lipid content, and the flexibility of the metabolism to adapt to external factors [2,31,32,33,34].

The significantly smaller increase in postprandial RQ, the lower carbohydrate oxidation, as well as the higher fat oxidation found with ISO consumption is in accordance with other studies. A body of literature shows a greater fat oxidation with LGI compared to HGI diets [13,35,36]. Several studies have confirmed these effects using ISO as a low-glycemic carbohydrate [9,10,12,13,19,35,36].

The shift in substrate oxidation with ISO presumably led to a larger decrease in waist circumference (WC) of about –1 cm. WC is used as a surrogate marker for visceral adipose tissue (VAT), which is closely linked to impaired glucose tolerance, peripheral insulin resistance, and the development of metabolic disorders [2,6,37,38]. Thus, our data indicate that consuming foods with ISO compared to conventional SUC may not only help to reduce body weight but also improve metabolic conditions. The data may be supported by previous studies which show a close correlation of GI with general and central obesity [39]. Moreover, a greater weight loss with LGI compared to HGI diets has been observed [40,41,42]. In addition, several studies have shown an improvement in metabolic risk factors, e.g., insulin resistance or a decreased prevalence for developing metabolic syndrome [16,19,43]. A study by Kahlhöfer et al. demonstrated that sugar-sweetened beverages containing ISO compared to a SUC–maltodextrin mixture preserved insulin sensitivity during an inactive phase in healthy, young men [19]. This highlights the beneficial effects of low-glycemic carbohydrates not only in overweight/obese or metabolically impaired persons, but also in healthy, normal weight individuals.

Even though participants were allowed to individually choose their dinner ad libitum (~800 kcal), the weight loss observed was more pronounced with ISO. Thus, the results indicate that when almost half of the daily meals contain ISO, positive metabolic effects may occur and compensate for the residual food intake. The property of LGI meals to improve postprandial metabolic response not only occurs directly after its consumption but also improves the postprandial glycemic and insulinemic response to the subsequent meal, which is described as the “second meal effect” [44,45]. 

Furthermore, it is known that incretins, i.e., GIP (glucose-induced insulinotropic peptide) and GLP-1 (glucagon-like peptide 1), which are strongly suggested to mediate metabolic responses, are secreted differently after ISO-containing LGI versus HGI meals. Studies in humans and rodents have shown an increased secretion of GLP-1 with ISO compared to that with SUC and, in turn, a reduced secretion of GIP [46,47]. This may be attributed to the underlying locations and modes of absorption which differ between both carbohydrates. Consequentially, different epithelial cells (for GLP-1 L cells, for GIP K cells) are stimulated, secreting the respective incretins. These have crucial effects on insulin secretion and action, and thus, over the long-term, also on insulin sensitivity and glucose tolerance [48,49]. Moreover, after 12 weeks of intervention, the present study found a significantly higher reduction in energy intake from baseline with ISO compared to that with SUC (*p* = 0.022). Hence, higher satiety induced by ISO may be assumed, which would be in accordance with the literature, demonstrating a reduced hunger or even reduced energy intake with LGI compared to HGI diets [50,51].

The strength of the present study is the randomized, double-blind, and controlled design, as well as the close follow-up of participants and the regular contact every 4 weeks over the study period. The chosen study period of 12 weeks was sufficient to observe body weight changes in overweight and obese individuals. An additional strength was the dietary intervention, which controlled for around half of the daily calories only. Thus, restrictions to the participants were kept to a minimum, which may have resulted in the high adherence to the protocol in both groups. Furthermore, potential confounding factors as present in other dietary intervention studies comparing LGI and HGI diets were prevented by providing comparable meals only differing in the test carbohydrates. 

One limitation of the study may be that the assessment of body composition was undertaken by air-displacement plethysmography, while more detailed information, e.g., from using DEXA (Dual-Energy X-Ray Absorptiometry) or MRI techniques, could have provided further useful insights into the mechanisms. Moreover, determination of further metabolic and hormonal parameters, such as insulin, would have provided additional elucidation of the mode of action. The participants´ liking of the test products could also have had an impact on energy intake, yet individual sensory acceptance was not assessed. 

## 5. Conclusions

Weight loss is associated with the improvement of components of the metabolic syndrome, for example, glycemic control, reduced body fat or improved blood lipids. The present weight loss intervention in overweight and obese individuals revealed that participants on an LGI diet with ISO showed a higher weight loss as well as fat mass loss compared to their counterparts in the SUC group. The higher overall decrease in EI as well as the enhanced fat oxidation at the expense of carbohydrates (i.e., reduction in postprandial RQ), which was observed concomitantly with the ISO intervention, may account for the beneficial effects. Our data hence suggest that ISO, a slow-release and low-glycemic sugar alternative, might be a useful tool to promote weight loss and thus improve metabolic risk factors. Additional beneficial effects of isomaltulose consumption on the maintenance of weight loss merit further examinations. 

## Figures and Tables

**Table 1:** Anthropometrics, body composition, and energy metabolism of subjects before, during, and after the 12-week intervention (mean ± SD).

	Isomaltulose				Sucrose			
	Baseline	Week 4	Week 8	Week 12	Baseline	Week 4	Week 8	Week 12
Females/males (n)	21/4 (25)	–	–	–	20/5 (25)	–	–	–
Age (yr)	40.2 ± 12	–	–	–	41.2 ± 12	–	–	–
**Anthropometrics and body composition**								
Weight (kg)	82.8 ± 13.3 ^a^	81.0 ± 13.2 ^b^	80.0 ± 12.4 ^c^	79.6 ± 12.5 ^c^	83.2 ± 12.6 ^a^	81.9 ± 12.2 ^b^	81.3 ± 11.8 ^b^	81.0 ± 11.7 ^b^
BMI (kg/m^2^)	29.7 ± 2.9 ^a^	29.0 ± 2.9 ^b^	28.7 ± 2.8 ^c^	28.6 ± 2.9 ^c^	29.1 ± 2.5 ^a^	28.7 ± 2.4 ^b^	28.5 ± 2.3 ^b^	28.5 ± 2.3 ^b^
WC (cm)	91.4 ± 10.2 ^a^	90.1 ± 9.9 ^a^	87.9 ± 8.8 ^a^	87.9 ± 7.3 ^a^	90.7 ± 7.5 ^a^	89.1 ± 8.1 ^a^	90.0 ± 6.3 ^a^	89.1 ± 7.5 ^a^
Fat mass (%)	38.9 ± 5.7 ^a^	38.0 ± 6.1 ^a,b^	38.2 ± 6.0 ^a,b^	37.0 ± 6.9 ^b^	40.1 ± 6.2 ^a^	39.3 ± 6.8 ^a^	39.9 ± 6.5 ^a^	39.3 ± 6.6 ^a^
Fat free mass (%)	60.9 ± 5.8 ^a^	61.7 ± 6.6 ^a,b^	61.8 ± 6.0 ^a,b^	63.0 ± 6.9 ^b^	59.9 ± 6.2 ^a^	61.0 ± 6.9 ^a^	60.3 ± 6.6 ^a^	61.0 ± 6.6 ^a^
**Energy intake and metabolism**								
Total EI (kcal/d)	2223 ± 486 *^,a^	1828 ± 397 ^b^	1719 ± 347 ^b^	1679 ± 306 ^b^	1900±372 *^,a^	1705 ± 344 ^a,b^	1583 ± 233 ^b^	1617 ± 245 ^b^
REE (kcal/d)	1538 ± 318 ^a^	1526 ± 353 ^a^	1480 ± 336 ^a,b^	1460 ± 333 ^b^	1502 ± 226 ^a^	1472 ± 223 ^a^	1467 ± 235 ^a^	1463 ± 241 ^a^
ppEE (kcal/d)	1781 ± 366 ^a^	1745 ± 336 ^a^	1652 ± 456 ^a^	1690 ± 291 ^b^	1800 ± 278 ^a^	1762 ± 233 ^a^	1749 ± 240 ^a^	1747 ± 238 ^a^
Fasting RQ	0.81 ± 0.06 ^a^	0.81 ± 0.06 ^a^	0.79 ± 0.05 ^a^	0.80 ± 0.05 ^a^	0.80 ± 0.06 ^a^	0.80 ± 0.05 ^a^	0.79 ± 0.05 ^a^	0.81 ± 0.06 ^a^
ΔRQ_pp-fasting_	0.04 ± 0.05 ^a^	0.03 ± 0.10 ^a^	0.05 ± 0.05 ^a^	0.06 ± 0.05 ^a^	0.07 ± 0.05 ^a^	0.07 ± 0.04 ^a^	0.07 ± 0.04 ^a^	0.07 ± 0.03 ^a^
iAUC_2h_ RQ	4.4 ± 4.9 ^a^	3.1 ± 10 ^a^	5.6 ± 6.5 ^a^	6.2 ± 6.5 ^a^	6.8 ± 7.9 ^a^	6.9 ± 6.8 ^a^	7.7 ± 8.2 ^a^	6.8 ± 6.6 ^a^
Total AUC_2h_ CO (kcal)	64 ± 34 ^a^	65 ± 30 *^,a^	62 ± 23 ^a^	68 ± 29 ^a^	74 ± 34 ^a^	93 ± 57 *^,a^	70 ± 31 ^a^	78 ± 36 ^a^
Total AUC_2h_ FO (kcal)	78 ± 51 ^a^	77 ± 47 ^a^	76 ± 40 ^a^	71 ± 44 ^a^	75 ± 36 ^a^	68 ± 31 ^a^	73 ± 37 ^a^	62 ± 32 ^a^

BMI, body mass index; CO, carbohydrate oxidation; EI, energy intake; FO, fat oxidation; iAUC, incremental area under the curve; ppEE, postprandial energy expenditure (calculated as the mean of the values at 30, 60, 90, and 120 min); REE, resting energy expenditure; RQ, respiratory quotient; WC, waist circumference. Significant within-group differences are characterized by values without a common superscript letter (repeated measures ANOVA: *p* < 0.05; Friedman test: *p* < 0.017). Significant between-group differences are marked with an asterisk* (independent sample t-test/Mann–Whitney U test: *p* < 0.05).

## Supplementary Materials

The following are available online at https://www.mdpi.com/2072-6643/11/10/2367/s1, Table S1: Meal composition of the energy-reduced study diets; Table S2: Macronutrient intakes at weeks 4, 8, and 12 compared to baseline (mean ± SD); Table S3: Changes in macronutrient intakes at weeks 4, 8, and 12 compared to baseline (mean ± SD); Table S4: Basal characteristics of blood pressure, blood glucose, and blood lipids at baseline and weeks 4, 8, and 12 (mean ± SD).
