# Supplementary material for: Changes in Weight and Substrate Oxidation in Overweight Adults Following Isomaltulose Intake During a 12-Week Weight Loss Intervention: A Randomized, Double-Blind, Controlled Trial
Source: Nutrients. 2019 Oct 4;11(10):2367. doi: 10.3390/nu11102367 (PMC6836138; doi:10.3390/nu11102367)
Supplement: Supplementary file 1 [file nutrients-11-02367-s001.pdf]

Table S1 Meal composition of the energy-reduced study diets.

Meal	Meal composition	Caloric value
**Breakfast**	Weetabix (shredded wheat biscuits) with semi-skimmed milk<br>+ 10g ISO or 10g SUC	204 kcal<br>+80 kcal
**1st Snack**	Vegetable/fruit	100 kcal
**Lunch**	Low-calorie sandwich<br>+ 250ml fruit drink containing<br>20g ISO or 20g SUC	298 kcal<br>+80 kcal
**2nd Snack**	Low-fat yogurt drink containing 10g ISO or 10g SUC	112 kcal
**Evening meal**	Ad libitum self-chosen diet. Participants were provided with healthy eating guidelines and a list of low energy dense foods.	~800 kcal

ISO, isomaltulose; SUC, sucrose.

Table 2. Macronutrient intakes at week 4, week 8 and week 12 compared to baseline (mean ± SD).

	Isomaltulose				Sucrose			
	**Baseline**	**Week 4**	**Week 8**	**Week 12**	**Baseline**	**Week 4**	**Week 8**	**Week 12**
Protein (g/day)	91.8 ± 25.3[a,*]	82.7 ± 13.8 [a,b]	78.2 ± 14.3 [a,b]	76.1 ± 14.1 [b]	71.8 ± 15.9 [a,*]	75.6 ± 13.3 [a]	73.7 ± 15.7 [a]	75.3 ± 13.6 [a]
Fat (g/day)	87.9 ± 25.3 [a,*]	55.8 ± 31.1 [b]	50.9 ± 21.7 [b]	47.9 ± 18.2 [b]	73.5 ± 22.2 [a,*]	49.7 ± 32.3 [b]	43.1 ± 11.5 [b]	40.9 ± 11.5 [b]
CHO (g/day)	262.5 ± 60.7 [a,*]	251.4 ± 46.4 [a]	240.8 ± 49.0 [a]	240.6 ± 44.2 [a]	232.1 ± 56.0 [a,*]	244.0 ± 38.9 [a]	226.0 ± 35.8 [a]	241.3 ± 39.3 [a]

CHO, carbohydrate; ISO, isomaltulose; SUC, sucrose. Significant within-group differences are characterized by values without a common superscript letter (dependent t test: $p < 0.05$). Significant between-group differences are marked with an asterisk *(independent sample t-test: $p < 0.05$).

Table S3 Changes in macronutrient intakes at week 4, week 8 and week 12 compared to baseline (mean ± SD).

	ISO			SUC		
	**Δwk4 – baseline**	**Δwk8 – baseline**	**Δwk12 – baseline**	**Δwk4 – baseline**	**Δwk8 – baseline**	**Δwk12 – baseline**
ΔProtein (g/day)	−9.1 ± 25.5	−13.6 ± 30.2	−15.7 ± 27.0 *	3.8 ± 20.4	−1.1 ± 24.1	3.4 ± 16.7 *
ΔFat (g/day)	−32.1 ± 34.1	−37.0 ± 29.3	−40.0 ± 25.8	−25.1 ± 26.2	−30.6 ± 19.1	−33.1 ± 18.3
ΔCHO (g/day)	−11.2 ± 67.9	−21.8 ± 63.4	−21.9 ± 64.7	11.8 ± 69.6	−11.2 ± 53.8	9.1 ± 56.3

CHO, carbohydrate; ISO, isomaltulose; SUC, sucrose. No within-group differences were observed (paired t-test: $p < 0.05$). Significant between-group differences are marked with an asterisk *(independent sample t-test: $p < 0.05$).

Table 4. Basal characteristics of blood pressure, blood glucose and blood lipids at baseline, week 4, week 8 and week 12 (mean ± SD).

	Isomaltulose				Sucrose			
	**Baseline**	**Week 4**	**Week 8**	**Week 12**	**Baseline**	**Week 4**	**Week 8**	**Week 12**
Systolic BP (mm Hg)	<u>121 ± 12 ^{a,*}</u>	119 ± 14 ^{a}	120 ± 12 ^{a}	119 ± 12 ^{a}	<u>129 ± 15 ^{a,*}</u>	122 ± 9 ^{b}	123 ± 14 ^{b}	123 ± 15 ^{b}
Diastolic BP (mm Hg)	81 ± 8 ^{a}	77 ± 12 ^{a}	78 ± 9 ^{a}	78 ± 9 ^{a}	84 ± 11 ^{a}	81 ± 11 ^{a}	83 ± 12 ^{a}	82 ± 13 ^{a}
FBG (mmol/L)	4.4 ± 0.4 ^{a}	4.5 ± 0.6 ^{a}	4.5 ± 0.7 ^{a}	4.4 ± 0.6 ^{a}	4.2 ± 0.5 ^{a}	4.2 ± 0.9 ^{a}	4.5 ± 0.6 ^{a}	4.4 ± 0.4 ^{a}
Cholesterol (mmol/L)	4.4 ± 1.1 ^{a}	4.4 ± 1.0 ^{a}	4.3 ± 1.0 ^{a}	4.6 ± 0.8 ^{a}	4.9 ± 1.3 ^{a}	4.6 ± 1.2 ^{a}	4.6 ± 1.0 ^{a}	4.6 ± 1.0 ^{a}
TG (mmol/L)	1.5 ± 0.5 ^{a,b}	1.4 ± 0.4 ^{a}	1.3 ± 0.5 ^{b,c}	1.2 ± 0.3 ^{c}	1.4 ± 0.6 ^{a}	1.7 ± 0.9 ^{a}	1.7 ± 1.2 ^{a}	1.6 ± 1.3 ^{a}

BP, blood pressure; FBG, fasting blood glucose; TG, triglycerides. Significant within-group differences are characterized by values without a common superscript letter (dependent t test: $p < 0.05$). Significant between-group differences are marked with an asterisk* (independent sample t-test/Mann-Whitney U test: $p < 0.05$).
